# De Novo Assembly and Discovery of Genes That Involved in Drought Tolerance in the Common Vetch

**DOI:** 10.3390/ijms20020328

**Published:** 2019-01-15

**Authors:** Yongqun Zhu, Qiuxu Liu, Wenzhi Xu, Jianhua Zhang, Xie Wang, Gang Nie, Li Yao, Hong Wang, Chaowen Lin

**Affiliations:** 1 Institute of Soil and Fertilizer Research, Sichuan Academy of Agricultural Sciences, Chengdu 610066, China; zyq80842@hotmail.com (Y.Z.); xuwenzhi_herb@126.com (W.X.); zjhu-11@163.com (J.Z.); wxowdj@126.com (X.W.); yaolisfri@163.com (L.Y.); wang.hongde163@163.com (H.W.); 2 Department of Grassland Science, Animal Science and Technology College, Sichuan Agricultural University, Chengdu 611130, China; sicauliuqiuxu@163.com (Q.L.); nieganggrass@hotmail.com (G.N.)

**Keywords:** common vetch, next-generation sequencing, differentially expressed genes, drought

## Abstract

The common vetch (*Vicia sativa*) is often used as feed for livestock because of its high nutritional value. However, drought stress reduces forage production through plant damage. Here, we studied the transcriptional profiles of common vetch exposed to drought in order to understand the molecular mechanisms of drought tolerance in this species. The genome of the common vetch has not been sequenced, therefore we used Illumina sequencing to generate de novo transcriptomes. Nearly 500 million clean reads were used to generate 174,636 transcripts, including 122,299 unigenes. In addition, 5313 transcription factors were identified and these transcription factors were classified into 79 different gene families. We also identified 11,181 SSR loci from di- to hexa-nucleotides whose repeat number was greater than five. On the basis of differentially expressed genes, Gene Ontology analysis identified many drought-relevant categories, including “oxidation-reduction process”, “lipid metabolic process” and “oxidoreductase activity”. In addition to these, Kyoto Encyclopedia of Genes and Genomes (KEGG) pathway analysis identified pathways, such as “Plant hormone signal transduction”, “Glycolysis/Gluconeogenesis” and “Phenylpropanoid biosynthesis”, as differentially expressed in the plants exposed to drought. The expression results in this study will be useful for further extending our knowledge on the drought tolerance of common vetch.

## 1. Introduction

The common vetch (*Vicia sativa*) is an annual, self-pollinated and diploid leguminous forage [1,2,3,4]. It is adaptable to different soil and climate and can fix nitrogen to improve soil structure. In addition to these qualities, the common vetch is nutritious to animals and is used in agriculture as feed, green manure and silage [5,6,7]. It is widely planted and used for agriculture in Turkey, Australia, New Zealand, China and other regions of the world [5,7].

Drought stress is one of the most common abiotic stresses that plants experience and cause cellular damage and secondary stresses, such as osmotic and oxidative stress and reduced membrane stability, which eventually leads to cell death by complex reaction [8]. Plants have evolved a variety of defense mechanisms and physiological responses to withstand drought, such as changes in signal transduction, metabolism and gene expression [9]. Although various breeding methods have been used to mitigate damages caused by drought stress in plants, genetic engineering is more effective than traditional breeding. However, genetic engineering requires identifying genes that are important for drought tolerance and understanding genes that are differentially expressed under drought stress is important for identifying these genes. Previous studies have identified genes that show a transcriptional response to drought stress in other higher plants [10].

Next generation sequencing allows for the generation of large-scale transcriptome data in both model and non-model species. Since Hegedus et al. [11] first used Solexa/Illumina′s Digital Gene Expression (DGE) system to study the transcriptome of zebrafish infected with Mycobacterium marinum, high-throughput RNA sequencing (RNA-Seq) and DGE technology have been widely used to identify plant genes, including those expressed in stress condition [12,13] and for other important agronomic traits.

Here, we aimed to identify genes involved in drought tolerance using Ilumina tag-sequencing and screening for differentially expressed genes (DEGs). We further validated DEGs using qPCR. To our knowledge, this is the first transcriptome resource for the common vetch. DEGs identified in this study will help to elucidate the common vetch′s molecular response mechanism to drought stress and serve as a reference for improving drought resistance in the common vetch through future genetic modifications.

## 2. Results and Discussion

### 2.1. Transcriptome Sequencing and De Novo Assembly

A total of 10 cDNA libraries from the control (0 days, 3 days, 5 days) and drought treated (3 days, 5 days) were generated and referred to as C0, C1, C2, D1 and D2. Each condition had two biological replicates and those were referred to as C0a, C0b and so forth. Overview of the sequencing and assembly results are listed in Table 1 and they have been deposited in the NCBI (National Center for Biotechnology Information) Short Read Archive (SRA: SRR8186820). More than 95.56% of the bases from the raw reads had Q value ≥ 20 (an error probability of 0.02%) and approximately 90% of the bases from the raw reads had Q value ≥ 30 (an error probability of 0.02%). The GC-content was between 42.46% and 42.98%. These reads were used for de novo assembly of the transcriptome.

After removing low-quality raw reads, there were 500 million clean reads, accounting for more than 95.96% of the raw reads. Trinity was used to generate 174,636 transcripts (Table 2) with an average length of 1124 bp and a N50 of 1991 bp. Of these, 122,299 were unigenes, where 23,874 unigenes were 200–500 bp, 30,990 unigenes were 500–1000 bp, 36,207 unigenes were 1–2 kb and the remaining 31,228 unigenes were > 2 kb (Table 2).

### 2.2. Functional Annotation and Pathway Assignment of Genes

All the assembled unigenes were searched against the Non-Redundant Protein Sequence Database (Nr), Nucleotide Sequence Database (Nt), Kyoto Encyclopedia of Genes and Genomes (KEGG), Swiss-Prot, Pfam, Gene Ontology (GO) and Clusters of orthologous groups for eukaryotic complete genomes (KOG) databases. A total of 102,106 unigenes were annotated, accounting for 83.48% of the unigenes (Table 3). A total of 16,574 (13.55%) unigenes were annotated in all seven databases. The number of unigenes with significant similarity to sequences in Nr, Nt, KEGG, Swiss-Prot, Pfam, GO and KOG databases were 90,190 (73.74%), 90,947 (74.36%), 37,056 (30.39%), 71,241 (58.24%), 65,975 (53.94%), 67,889 (55.51%) and 27,407 (22.4%), respectively (Table 3).

**Figure 1:**
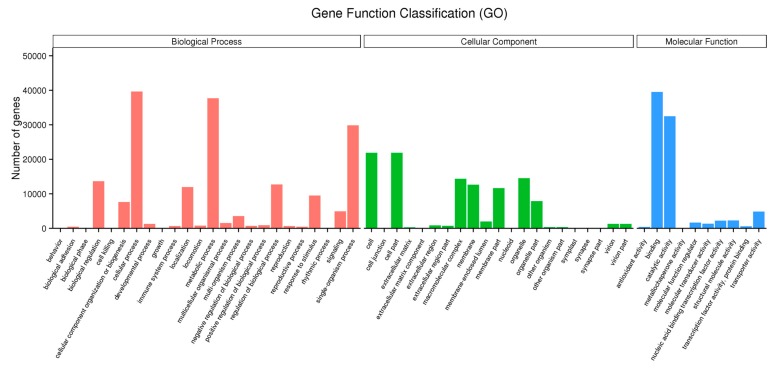
Gene Function Classification of the assembled unigenes. Unigenes with BLAST hits were classified into three major categories and 55 sub-categories in GO. The Y-axis represents the number of genes in each category.

Using GO classification, 67,889 unigenes were classified into three functional categories: biological process, cellular component and molecular function (Figure 1, Table S1). In the biological process category, unigenes clustered into 24 classifications where the largest subcategory was “cellular process” and the second largest subcategory was “metabolic process”. In the cellular component category, unigenes were clustered into 21 classifications and most belonged to the subcategories “cell” and “cell part”. In the molecular function category, unigenes were divided into 10 classifications with the most represented subcategories being “binding” and “catalytic activity”.

**Figure 2:**
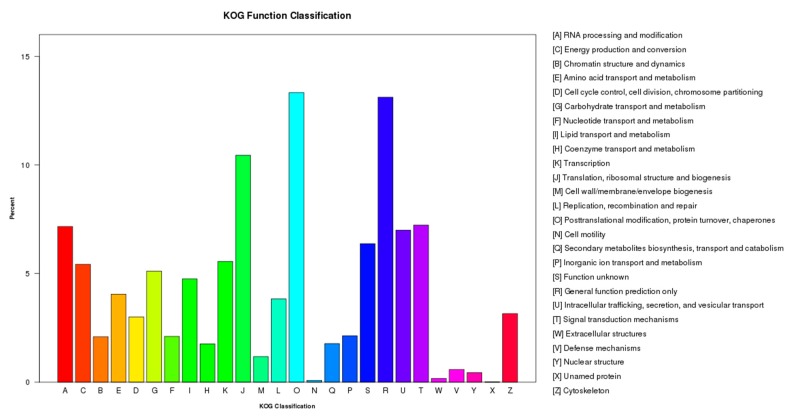
KOG Function Classification of unigenes involved in drought tolerance in the common vetch. Genes clustered into 26 groups, where the Y-axis indicates the number of unigenes in each group as annotated on the right.

Prediction of gene function was conducted by searching unigene sequences were against the KOG database (Figure 2, Table S2) and we found that 27,407 unigenes clustered into 26 groups in the KOG database. The largest group was group O “Posttranslational modification, protein turnover and chaperones” (3653, 13.33%), followed by group R “General function prediction only” (3595, 13.12%), group J “Translation, ribosomal structure and biogenesis” (2862, 10.44%), group T “Signal transduction mechanisms” (1981, 7.23%), group A “RNA processing and modification” (1963, 7.16%), group U “Intracellular trafficking, secretion and vesicular transport” (1918, 7.00%), group S “Function unknown” (1747, 6.37%), group K “Transcription” (1523, 5.56%), group C “Energy production and conversion” (1486, 5,42%), group G “Carbohydrate transport and metabolism” (1400, 5.11%), group I “Lipid transport and metabolism” (1303, 4.75%), group E “Amino acid transport and metabolism” (1109, 4.05%), group L “Replication, recombination and repair” (1050, 3.83%), group Z “Cytoskeleton” (865, 3.16%), group D “Cell cycle control, cell division and chromosome partitioning” (823, 3.00%), group B “Chromatin structure and dynamics” (572, 2.09%), group Q “Secondary metabolites biosynthesis, transport and catabolism” (486, 1.77%), group H “Coenzyme transport and metabolism” (483, 1.76%) and group M “Cell wall/membrane/envelope biogenesis” (322, 1.17%). Less than 1% of the unigenes were assigned categories to “Defense mechanisms”, “Nuclear structure”, “Extracellular structures”, “Cell motility” and “Unnamed protein”.

To explore the potential function of the unigenes in the common vetch, the biochemical pathways and functions associated with the unigenes were assigned by KEGG. A total of 37,056 were assigned to five KEGG biochemical pathways (Table S3): Cellular processes (1808), environmental information processing (1345), genetic information processing (7966), metabolism (16,705) and organismal systems (1202). The largest group was metabolic pathways and many of the unigenes within this group were associated with carbohydrate metabolism (3349), overview (2254), amino acid metabolism (1991) and lipid metabolism (1799). Genetic information processing was the second largest group, including genes involved in translation (3377), folding, sorting and degradation (2517), transcription (1342) and replication and repair (730). Pathways related to cellular processes, environmental information processing and organismal systems were also well represented. These results provide a valuable resource for investigating metabolic pathways in the common vetch.

### 2.3. Transcription Factors

Transcription factors (TFs) are important upstream regulatory proteins that regulate the plant’s responses to abiotic and biotic stress and were overexpressed to enhanced the plant resistance [14,15,16,17,18,19]. In the common vetch transcriptome, we identified 5313 TFs that were classified into 79 different common families (Table S4). The largest group of TFs was the MYB family (415, 7.81%), followed by bHLH (315, 5.93%), Orphans (245, 4.61%), AP2-EREBP (245, 4.61%), C3H (244, 4.59%) and WRKY (235, 4.42%). These results are similar to the *Chrysanthemum morifolium* transcriptome, where largest TF group is MYB, followed by Zinc finger, AP2/EREBP and HB families [20], as well as the ramie transcriptome where the largest TF groups belonged to the bZIP, MYB, AP2/ERF and WRKY families [21]. These results imply that bZIP, MYB, AP2/ERF and WRKY are TF superfamilies in plants. At the same time, on the one hand, we found that the MYB, bHLH, C3H, WRKY and bZIP families that are well-known in stress tolerance in plants were identified. Members of MYB (Cluster-8152.4887 and Cluster-8152.51806), bHLH (Cluster-8152.52494 and Cluster-8152.29098), C3H (Cluster-8152.48221 and Cluster-8152.68994), WRKY (Cluster-8152.31165), bZIP (Cluster-8152.63327) family are always up-regulation under drought stress, suggesting were positive regulation mechanism in the common vetch. On the other hand, there are a few members of the transcription factor family that have been down-regulated, such as member of bHLH (Cluster-8152.61311). The results indicate that TFs respond to drought stress in a variety of mechanisms and it is similar to what was found in *Zea mays ssp. mexicana* L. [22].

### 2.4. SSR Identification

Simple sequence repeats (SSRs) have much higher levels of polymorphisms than most other marker systems due to their codominance, hypervariability, high reproducibility and abundance in eukaryotic genomes. and we identified expression sequence tags- simple sequence repeats (EST-SSRs) in the transcriptome of the common vetch by analyzing the assembled contig templates. We identified a total of 24,914 distant SSR loci were identified (Table S5) and among these loci, SSR loci repeat number greater than five accounted for 11,181. Most of these satellites were mono-nucleotide motifs with more than 10 repeats, accounting for 13,733 (55.12%). AG/CT was the most frequent di-nucleotide SSR repeat and accounted for 2896 and AAG/TCC was the most frequent tri-nucleotide SSR repeat and accounted for 1284 (Table S5). Similarly, AG/CT is the most common di-nucleotide SSR repeat in *Sorghum sudanense* [23] and this may be due to the similar drought stress experienced by the two plants. Similarly, AAG/CTT is the most frequent tri-nucleotide SSR repeat in *Ammopiptanthus mongolicus* [24] and *Sophora moorcroftiana* [25], this is likely because the common vetch, A. *mongolicus* and S. *moorcroftiana* are all legumes that share similar genomic characteristics.

### 2.5. Differentially Expressed Genes under Drought Stress

**Figure 3:**
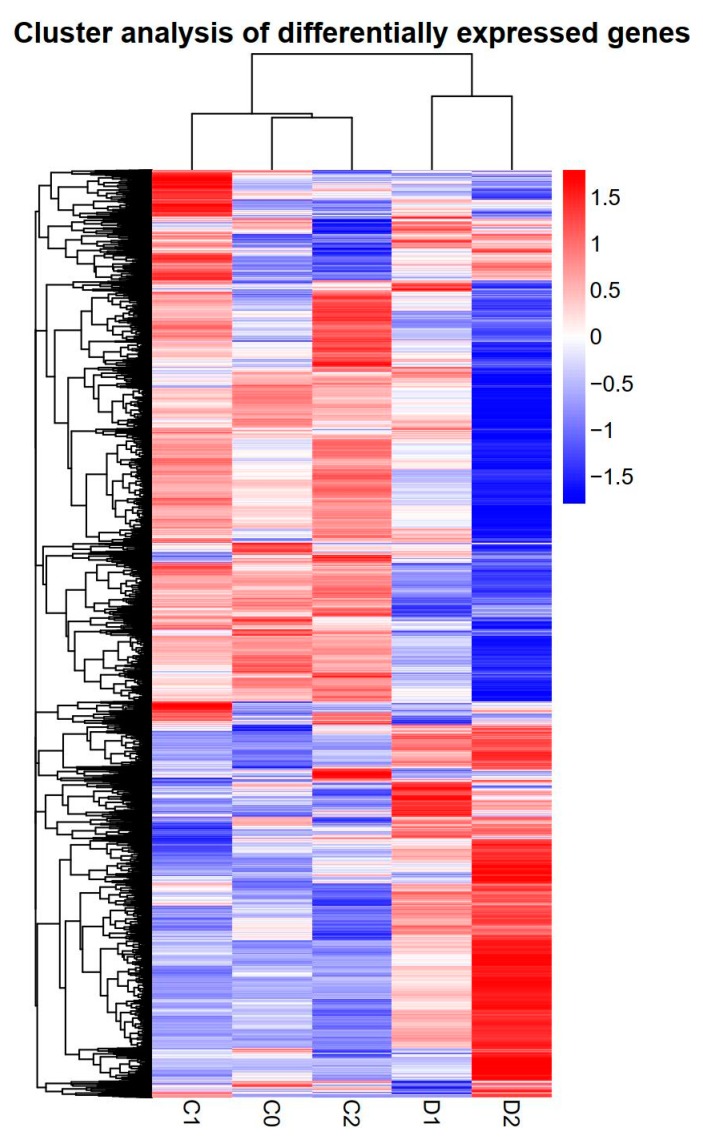
Cluster analyses of DEGs. The expression levels were log10 transformed and high levels of expression are indicated in red and low expression is indicated in blue.

**Figure 4:**
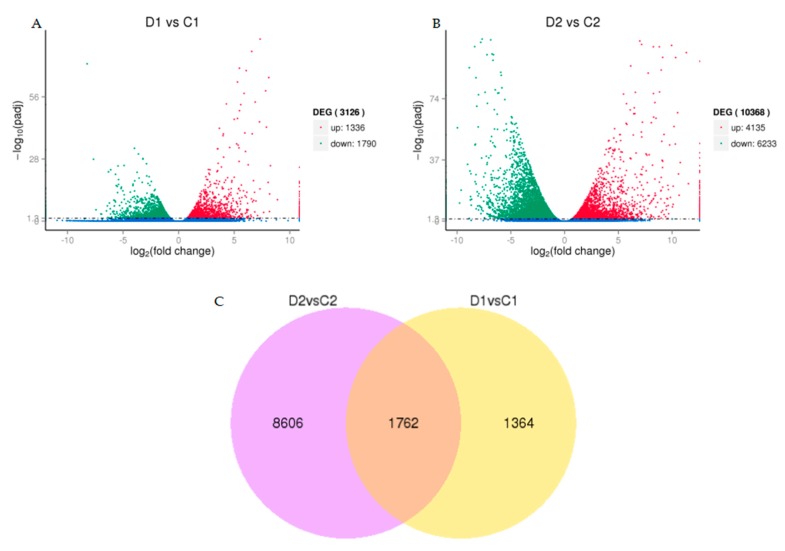
(**A**) Comparison of expression patterns of differential unigenes identified between D1 (drought 3 days) and C1 (control 3 days). (**B**) Comparison of expression patterns of differential unigenes identified between D2 (drought 6 days) and C2 (control 6 days). (**C**) Venn diagram showing DEGs across two comparisons (D2 versus C2 and D1 versus C1).

We found a high number of unigenes with differential expression in drought-treated samples. We identified differentially expressed genes (DEGs) with a *p* value-adjusted (padj) < 0.05 cut-off, conducted hierarchical clustering of the DEGs. The resulting gene expression profiles of the control and drought treated samples were highly divergent (Figure 3). We discovered 3126 and 10368 genes when comparing D1 versus C1 and D2 versus C2 (Figure 4A–B), respectively. A total of 1762 genes overlapped with those of D1 versus C1 and D2 versus C2 (Table S6), indicating that a shared set of genes was involved in response to drought stress at different time points (Figure 4C).

### 2.6. Functional Classification of the Drought-Responsive Stress Genes using Gene Ontology Analysis

**Figure 5:**
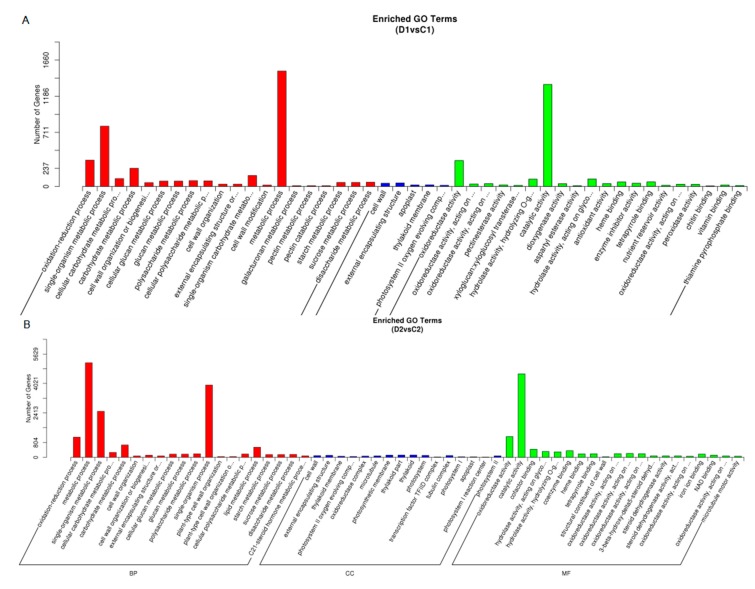
GO classifications of DEGs for (**A**) D1 (drought 3 days) versus C1 (control 3 days) and (**B**) D2 (drought 6 days) versus C2 (control 6 days). The Y-axis represents the number of DEGs in a category.

We next analyzed DEGs using GO analysis gain an understanding of the function of the DEGs. In the D1 and C1 comparing, there were 1336 up-regulated and 1790 down-regulated DEGs and the significantly overrepresented GO terms were “single-organism metabolic process” and “metabolic process” in the Biological Process category and “catalytic activity” in the Molecular Function category (Figure 5A). When comparing D2 C2, there were 4135 up-regulated and 6233 down-regulated DEGs and significant overrepresentation was found in “metabolic process” and “single-organism process” subcategories in the Biological Process category and “catalytic activity” subcategory in the Molecular Function category (Figure 5B). Overall, the results suggest that “single-organism metabolic process”, “single-organism process”, “metabolic process” and “catalytic activity” were strongly affected in samples treated with drought stress, which likely led to a strong metabolic response.

There was also an enrichment of DEGs categorized as “oxidation-reduction process” and “oxidoreductase activity”, which are commonly observed categories in drought-treated plants, suggesting that our drought treatment was effective. Other enriched categories included “carbohydrate metabolic process”, “lipid metabolic process”, “cofactor binding” and “coenzyme binding”, similar to previously published transcriptomes of high plants [23,25] and these results implicate that the interaction of different metabolic pathways is important for drought-response in plants.

### 2.7. KEGG Pathway Analysis of DEGs in Plants Exposed to Drought Conditions

**Figure 6:**
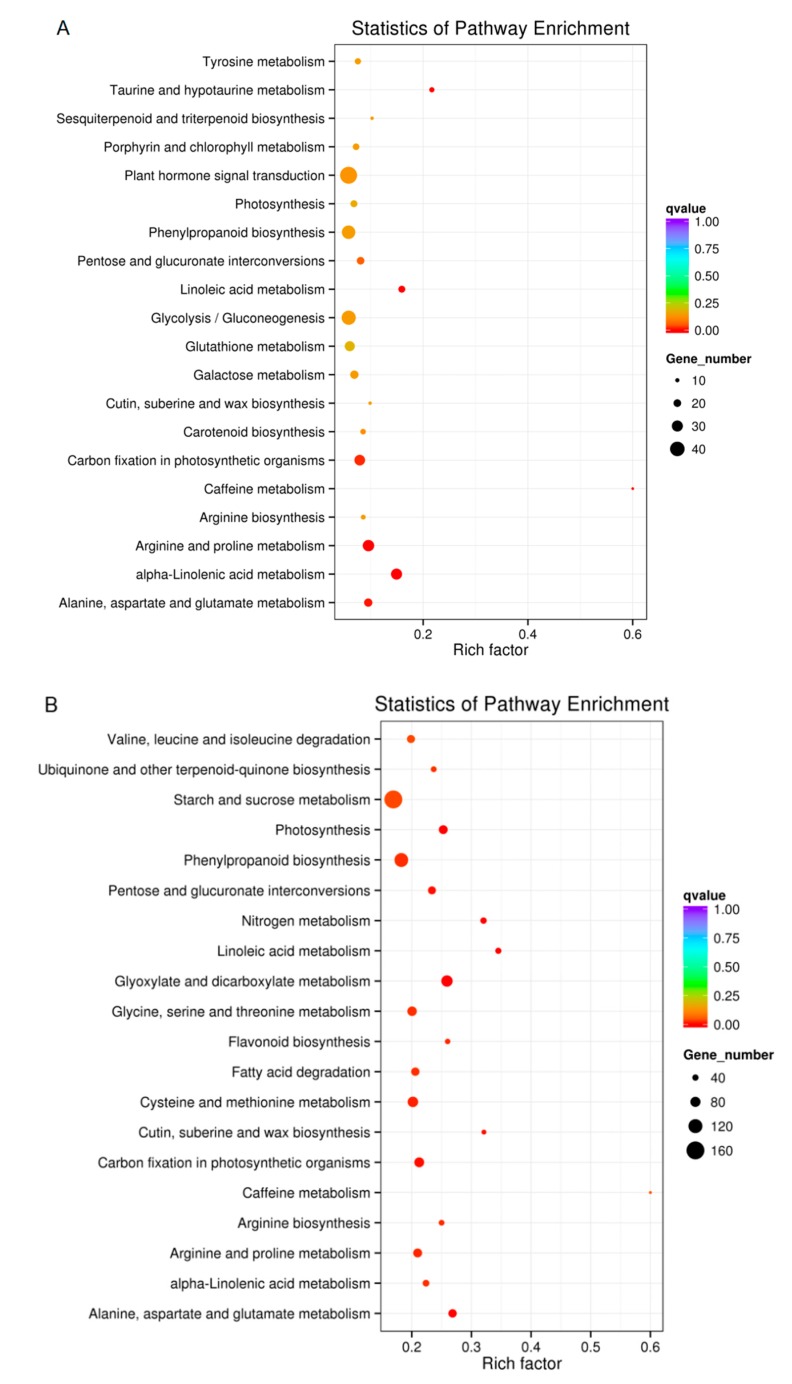
KEGG enrichments of annotated DEGs in (**A**) D1 (drought 3 days) versus C1 (control 3 days) and (**B**) D2 (drought 6 days) versus C2 (control 6 days). The Y-axis denotes the KEGG pathway and the X-axis denotes the Rich factor. A low q value is represented in red.

To determine whether the drought stress-responsive genes belonged to specific pathways, DEGs were searched against the KEGG database. The top 20 enriched pathways are listed in Figure 6. Comparisons between D1 and C1 showed that DEGs were enriched in “Plant hormone signal transduction”, “Glycolysis/Gluconeogenesis” and “Phenylpropanoid biosynthesis” (Figure 6A, Table S7). When comparing D2 and C2, the DEGs were enriched in “Starch and sucrose metabolism”, “Phenylpropanoid biosynthesis” and “Glyoxylate and dicarboxylate metabolism” (Figure 6B, Table S8). In general, these data indicate that drought stress affects “Plant hormone signal transduction”, “Glycolysis/Gluconeogenesis” and “Phenylpropanoid biosynthesis” in the common vetch.

Under abiotic stress, plants process information from the environment through signaling pathways to activate adaptive responses [26]. Histidine-containing phosphotansfer proteins (HPTs) are involved in the cytokinin transduction pathway [27] and cytokinin activity plays an important role in the plant’s response to salt, osmotic and drought stress [28]. Here, we found that HPTs (Cluster-8152.101072) were only expressed under drought stress with FC_D2_ vs. _D1_ = 1.43 and it may be involved in the common vetch’s resistance to environmental stress.

Changes in glycolysis and gluconeogenesis are common when plants respond to abiotic stress. Glycolysis is an important metabolic pathway that regulates carbohydrate metabolism and drought stress changes in sucrose and amino acid contents of plants [29]. DEGs in our analysis contained enzymes involved in glycolysis and glyconeogenesis and this is consistent with drought-mediated photosynthetic carbon metabolism [30,31]. In this study, the results show that the expression of some key enzymes in glycolysis/gluconeogenesis metabolism have changed under drought stress. For instance, putative phosphoglycerate mutase (PGAM), is regulated by an identified miRNA involved in the glycolysis pathway [32]. When water is insufficient, PGAM levels decrease [33,34]. We found that PGAM (Cluster-8152.18831) was significantly down-regulated under drought stress in the common vetch, suggesting that this gene may contribute to drought tolerance.

Signal transduction participate in numerous processes and has many pathways such as MAPK signaling pathway [35], Calcium signaling pathway [36], cAMP signaling pathway [37] and Plant hormone signal transduction [38,39]. In particular, plant hormone signal pathways, are extremely vital for plant development, growth, differentiation and adaptation to environmental stresses [40,41,42]. In this study, the results show that the expression of some key enzymes genes in plant hormone signal transduction were significantly up-regulated under drought stress. For example, protein phosphatase 2C (PP2C), have been shown to be key regulators of abscisic acid (ABA) signaling pathways, which regulate plant growth and development as well as tolerance to adverse environmental conditions [43]. The results showed that PP2C (Cluster-8152.72288) was significantly up-regulated (7.8251-fold) under drought stress, indicating this gene positively regulated the ABA signaling pathways and thus improved the drought resistance of plants.

Phenylpropanoids is a group of plant secondary metabolites derived from phenylalanine and are involved in differentiation and the protection of plant tissues against environment stresses [44]. Serine carboxypeptidase-like (SCPL) is a protease belonging to a family of hydrolases and is involved in the processing, modifying and degrading polypeptides and proteins during growth and development of plants [45,46,47,48]. As expected, we found that SCPL (Cluster-8152.36781) was significantly up-regulated under drought treatment, indicating that SCPL enhances drought resistance in the common vetch. Cinnamate 4-hydroxylase (C4H) is one of the most abundant P450s in plant [49] and is the key enzyme of the core reaction of the general phenylpropanoid pathway [44,50]. By comparing gene expression levels in drought and control conditions, we found that two candidate C4H genes, including Cluster-8152.77535 and Cluster-8152.50375, were significantly up-regulated. The results suggested that C4H protein participates in phenylpropanoid pathway to improve plants adaptation to the environment, as demonstrated by Yannick, B. [51].

### 2.8. Quantitative Real-Time-PCR Validation of DEGs from RNA-Seq

**Figure 7:**
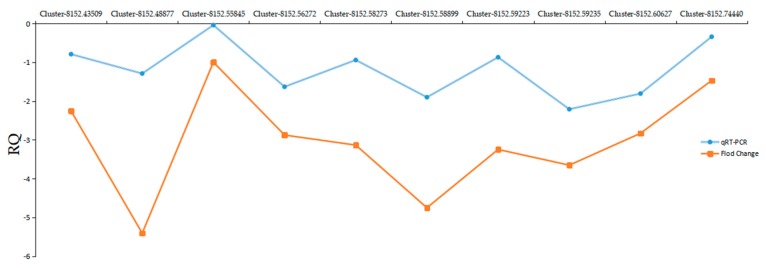
Unigene expression tendencies in both transcriptome and qRT-PCR analysis. The X-axis shows the different unigenes and the Y-axis represents expression in drought condition relative to the control. The numbers shown above the graphs indicated the fold changes for each unigene in the drought treatment relative to control conditions.

To confirm the gene expression data, 10 DEGs were randomly chosen for qRT-PCR analysis. The selected DEGs were all significantly down-regulated in drought-treated plants. The gene expression trends were similar in both the transcriptome and qRT-PCR data (Figure 7), validating our RNA-Seq data and DEG analysis.

## 3. Materials and Methods

### 3.1. Plant Material and Drought Treatment

The surface of *Vicia sativa* seeds were sterilized with 75% ethanol for 5 min and rinsed with sterile distilled water. Seeds were germinated in plastic pots with 10 g seeds per pot (20 cm length, 15 cm width and 8 cm deep) filled with sterilized quartz. Pots were kept in a controlled growth chamber at the Sichuan Agricultural University in Chengdu (30°42′ N, 103°51′ E; Chengdu Wenjiang, Sichuan, China) and the chamber was set to a 12 h photoperiod cycle, 19 °C/15 °C day/night temperature and 500 μmol photons m^−2^ s^−1^ photosynthetic active radiation (PAR) with a relative humidity of 75%. Three-day-old seedlings were irrigated with full strength Hoagland’s solution instead of distilled water, until the first leaf was expanded at about 13 cm high. Drought stress was imposed by 25% (*w*/*v*) polyethylene glycol (PEG) 6000 dissolved in Hoagland’s solution for five days and control plants were treated with Hoagland’s solution without PEG. Each treatment was performed in four independent replicates. Whole plants were collected at 0d as control (C0), 3d and 5d for control (C1 and C2), 3d and 5d for drought treatment (D1 and D2). Two independent biological replicates from each treatment were used for transcriptome sequencing.

### 3.2. RNA Extraction

Total RNA was extracted from the whole plant samples using the Trizol reagent (TransGen, Beijing, China) following the manufacturer’s instructions. Total RNA quality was first monitored using 1% agarose gels. RNA purity was tested using the NanoPhotometer^®^ spectrophotometer (IMPLEN, Palo Alto, CA, USA), concentration of the RNA was measured using the Qubit^®^ RNA Assay Kit in a Qubit^®^ 2.0 Fluorometer (Life Technologies, Carlsbad, CA, USA) and RNA integrity was assessed using the RNA Nano 6000 Assay Kit of the Agilent Bioanalyzer 2100 system (Agilent Technologies, Santa Clara, CA, USA).

### 3.3. Library Preparation for Transcriptome Sequencing

To construct the transcriptome library, 1.5 µg RNA per sample was used as input for each sequencing library. Sequencing libraries were generated using NEBNext^®^ Ultra™ RNA Library Prep Kit for Illumina^®^ (NEB, San Diego, CA, USA) following the manufacturer’s protocol and index codes were added to attribute sequences to each sample as follows. mRNA was purified from total RNA using poly-T oligo-attached magnetic beads and fragmentation was carried out using divalent cations under elevated temperatures in the NEBNext First Strand Synthesis Reaction Buffer (5X). First strand cDNA was synthesized using random hexamer primers and M-MuLV Reverse Transcriptase (RNase H-). Second strand cDNA synthesis was performed using DNA Polymerase I and RNase H and the remaining overhangs were converted into blunt ends via exonuclease/polymerase activities. After adenylation of the 3′ ends of the cDNA, NEBNext Adaptors with hairpin loop structures were ligated to prepare for hybridization. In order to select for 150–200 bp cDNA fragments, the library fragments were purified with the AMPure XP system (Beckman Coulter, Pasadena, CA, USA). Then 3 μL USER Enzyme (NEB, USA) was used with the size-selected and adaptor-ligated cDNA at 37 °C for 15 min and then incubated at 95 °C for 5 min before PCR. The PCR was performed with Phusion High-Fidelity DNA polymerase, Universal PCR primers and Index (X) Primer. PCR products were purified using the AMPure XP system (Beckman Coulter, Pasadena, CA, USA) and library quality was assessed on the Agilent Bioanalyzer 2100 system (Agilent Technologies, Santa Clara, CA, USA). Index-coded samples were clustered on a cBot Cluster Generation System (Illumina, San Diego, CA, USA) using TruSeq PE Cluster Kit v3-cBot-HS (Illumina, San Diego, CA, USA) following the manufacturer’s protocol. Once samples were clustered, libraries were sequenced on an Illumina Hiseq platform to generate paired-end reads.

### 3.4. Sequence Read Mapping, Assembly and SSR Detection

The raw data, or raw reads, in FASTQ format were processed through in-house Perl scripts. Clean data or clean reads were obtained by removing reads containing the adapter or ploy-N sequences, as well as removing low quality reads from the raw data. At the same time, Q20, Q30, GC-content and sequence duplication level of the clean data were calculated. All the downstream analyses were conducted on clean data that were of high quality. The transcriptome was assembled with the clean reads using Trinity [52] at default settings and with min_kmer_cov set to 2 by default. SSRs in the transcriptome were identified using MISA (http://pgrc.ipk-gatersleben.de/misa/misa.html). Protein coding sequences (CDS) of the assembled unigenes were predicted using BLAST and then EST Scan (E value < 10–5) [53].

### 3.5. Gene Expression Quantification and Differential Expression Analysis

Gene expression levels were estimated by RSEM (http://deweylab.github.io/RSEM/) [54] for each sample. The clean data were mapped back onto the assembled transcriptome and read count for each gene was obtained from the data mapped onto the transcriptome. To identify differentially expressed genes (DEGs), differential expression analysis of two groups was performed using the DESeq R package (1.10.1) (http://www.bioconductor.org/packages/release/bioc/html/DESeq.html). DESeq determines differential expression in digital gene expression data using a model based on a negative binomial distribution. The *p* values of the DESeq analyses were adjusted using the Benjamini and Hochberg’s approach to control for the false discovery rate. Genes with an adjusted *p*-value < 0.05 were assigned as differentially expressed.

### 3.6. Functional Annotation

Gene Ontology (GO) enrichment analysis of the DEGs was implemented by the GOseq R packages based Wallenius non-central hyper-geometric distribution [55], which can adjust for gene length bias in DEGs.

KEGG [56] is a resource for identifying high-level functions and utilities of the biological system, such as the cell, the organism and the ecosystem, from molecular-level information (http://www.genome.jp/kegg/). We used KOBAS [57] to test for the statistical enrichment of DEGs in KEGG pathways.

The DEGs were searched against the genomes of a related species using BLASTx and the protein-protein interactions (PPI) were tested using the STRING database (http://string-db.org/). The PPI of DEGs were visualized in Cytoscape [58].

### 3.7. Quantitative Real-Time-RCR Analysis

In order to validate the RNA-Seq data, 10 genes were randomly selected analyzed by qRT-PCR normalized to a reference gene (GAPDH; Table S9). RNA was isolated from relevant samples as described above. In a fluorescence quantitative PCR tube (TLS-0851; Bio-Rad), 2 µL of cDNA (30 ng/µL), 1.5 µL of reverse primer (10 µmol/L), 1.5 µL of forward primer (10 µmol/L), 10 µL 2 × SYBR Premix Ex Taq (5 U/µL) and 5 µL of ddH_2_O were added to a total volume of 20 µL. PCR was conducted using three biological replicates tested over three technical replicates. The amplification procedure was as follows: 95 °C for 30 s, followed by 95 °C for 5 s and 64 °C for 30 s, repeated 40 times, followed by an extension phase from 60 °C to 95 °C, where the temperature for each cycle increased by 0.5 °C for 5 s to obtain Tm and fluorescent signals for the melting curve. To determine the relative fold change for each sample in each experiment, the Ct value for the reference gene and candidate genes were calculated using the Ct method.

## 4. Conclusions

Here, we used RNA-Seq to generate the common vetch transcriptome and analyze changes in gene expression under drought stress. Based on the assembled de novo transcriptome, 3126 and 10,368 genes were discovered at three days and five days after inducing drought stress, respectively. The KEGG pathway analysis uncovered ‘Plant hormone signal transduction,’ ‘Glycolysis/Gluconeogenesis’ and ‘Phenylpropanoid biosynthesis’ as the important pathways associated with response to drought. We also developed new genetic markers, including SSRs, which can further be used for genetic studies in the common vetch.

## Figures and Tables

**Table 1:** Overview of the sequencing.

Gategory	C0		C1		C2		D1		D2		Total
	C0a	C0b	C1a	C1b	C2a	C2b	D1a	D1b	D2a	D2b	
Raw reads	50,421,376	52,941,226	54,578,760	46,387,122	54,257,670	44,592,212	54,615,492	54,797,818	54,777,004	47,989,120	515,357,800
Clean reads	483,648,36	50,722,480	52,325,294	44,492,586	52,050,600	42,819,464	52,423,694	52,648,460	52,609,898	46,059,876	494,517,188
Accounting	95.92%	95.81%	95.87%	95.92%	95.93%	96.02%	95.99%	96.08%	96.04%	95.98%	95.96%
Clean bases (G)	7.25	7.61	7.85	6.67	7.81	6.42	7.86	7.9	7.89	6.91	74.17
Q20 (%)	95.97	96.03	95.89	95.59	95.75	95.63	95.71	95.67	95.61	95.56	-
Q30 (%)	90	90.12	89.89	89.31	89.61	89.34	89.53	89.4	89.34	89.25	-
GC (%)	42.85	42.98	42.61	42.63	42.66	42.68	42.61	42.83	42.51	42.46	-
Error (%)	0.02	0.02	0.02	0.02	0.02	0.02	0.02	0.02	0.02	0.02	-

The control (0 days, 3 days, 5 days) and drought treated (3 days, 5 days) were generated and referred to as C0, C1, C2, D1 and D2. Each condition had two biological replicates and those were referred to as a, b and so forth.

**Table 2:** Summary statistics of the common vetch transcriptome assemblies.

Nucleotides Length (bp)	Transcripts	Unigenes
200–500	75,068	23,874
500–1000	32,116	30,990
1000–2000	36,224	36,207
>2000	31,228	31,228
Total	174,636	122,299
Minimal length	201	201
Maximal length	16,722	16,722
Median length	650	1142
Average length	1124	1483
N50	1991	2127
N90	442	722

**Table 3:** Unigene information annotated in different databases.

Public Database	Number of Unigenes	Percentage (%)
Annotated in Nr	90,190	73.74
Annotated in Nt	90,947	74.36
Annotated in KEGG	37,056	30.29
Annotated in Swiss-Prot	71,241	58.25
Annotated in Pfam	65,975	53.94
Annotated in GO	67,889	55.51
Annotated in KOG	27,407	22.4
Annotated in all Databases	16,574	13.55
Annotated in at least one Database	102,106	83.48
All assembled Unigenes	122,299	100

## Supplementary Materials

Supplementary materials can be found at http://www.mdpi.com/1422-0067/20/2/328/s1.

## Abbreviations

GO	Gene Ontology
KEGG	Kyoto Encyclopedia of Genes and Genomes
DGE	Digital Gene Expression
DEGs	Differentially Expressed Genes
TFs	Transcription factors
HPTs	Histidine-containing phosphotansfer proteins
PGAM	Putative Phosphoglycerate mutase
SCPL	Serine Carboxypeptidase-like
